# Weight gain in type 1 diabetes during the SARS-CoV-2 pandemic. Does lockdown affect the metabolic control of pediatric patients?

**DOI:** 10.3389/fendo.2022.991269

**Published:** 2022-10-12

**Authors:** Agnieszka Zubkiewicz-Kucharska, Beata Wikiera, Anna Noczyńska

**Affiliations:** Department of Pediatric Endocrinology and Diabetology, Wrocław Medical University, Wrocław, Poland

**Keywords:** BMI, metabolic control, type 1 diabetes, COVID-19 lockdown, children

## Abstract

**Background and aims:**

Due to the severe acute respiratory syndrome coronavirus 2 pandemic, governments of many countries decided to implement lockdowns, which included school closures. This major lifestyle change also applied to people with diabetes. The aim of this paper was to analyze how the COVID-19 pandemic and related restrictions influenced the metabolic compensation of diabetes in the pediatric population.

**Methods:**

Patients with type 1 diabetes (T1D), treated by one therapeutic team, who in 2020 and 2021 paid at least two in-person visits in the outpatient clinic, were included in the study. The time in range (TIR) and HbA1c, as well as the total daily dose (TDD) of insulin and BMI from the visit before the announcement of the pandemic restrictions (March 2020) and during the lockdown (second visit after 6 months) and within the period of loosened restrictions (two visits in 2021) were analyzed.

**Results:**

A total of 185 patients with T1D were included in the study (96 boys), aged 2–18 years (11.5 ± 3.5); 135 of them (72.9%) use CSII and 142 (76.8%) use CGM or FGM. During the first months of the studied period, despite comparable (p>0.05) TIR (57.5 ± 21.4% *vs*. 59.9 ± 20.5%), improvement of HbA1c was noticed (7.9 ± 1.6% *vs*. 7.5 ± 1.4%, p=0.0336), whereas in the following months, both HbA1c and TIR were comparable. Also, the TDD increased significantly (from 37.3 ± 18.9 units/day on the first visit up to 46.8 ± 22.7 units/day on the last visit, p=0.0003); however, TDD/kg remained constant (p>0.05) (0.8 ± 0.2 units/kg/day *vs*. 0.8 ± 0.3 units/kg/day) possibly due to an increased BMI (19.1 ± 3.7 kg/m^2^ *vs*. 20.9 ± 4.1 kg/m^2^, p=0.0001). The percentage of basal insulin in the TDD remained stable (p>0.05) (39.7 ± 11.3% *vs*. 39.3 ± 13.6%). Furthermore, a significant (p=0.0001) change in the BMI percentile was noticed [from 58.9 ± 26.2 percentiles (%iles) before lockdown *vs*. 64.6 ± 26.0%iles on the second visit]. However, the BMI percentile returned to baseline (58.1 ± 28.4%iles) at the visit at the end of the observation period.

**Conclusions:**

The parameters of metabolic control in pediatric patients with T1D during the pandemic period remained stable; however, weight gain and an increase in daily insulin dose have been observed, possibly due to reduced physical activity.

A novel coronavirus [severe acute respiratory syndrome coronavirus 2 (SARS-CoV-2)], which had caused a cluster of pneumonia in the Chinese province of Hubei in late 2019, spread throughout the world rapidly. On 11 March 2020, the World Health Organization (WHO) declared a pandemic of the disease induced by this virus—coronavirus disease 2019 (COVID-19).

SARS-CoV-2 is spread by droplet transmission. As it is a new pathogen for humanity, in early 2020, vaccination against this virus was not available. Therefore, it can be concluded that only the minority of the human population who suffered from COVID-19 had developed adaptive immunity against SARS-CoV-2, whereas the majority were susceptible to infection. The reproduction ratio, R0, of SARS-CoV-2 was estimated to be between 2 and 3.5. This means that every patient with COVID-19 infects, on average, two to three people, and, as so, the number of affected people doubles every week (1).

For obvious reasons, before the development of vaccination against this virus, the only effective method to reduce the spread of COVID-19 was social distancing. In order to reduce viral exposure and possible contamination, social isolation, “stay at home” orders, followed by lockdowns or semi-lockdowns were imposed in many countries, including Poland. To reduce public gatherings, many public spaces were closed, including gyms and swimming pools. Moreover, distance learning in schools and universities was introduced, which globally affected more than 90% of students (2). As a consequence of schools’ closure, limitation of participation in sports activities and reduction of children’s physical activity, as well as an increase in sedentary behavior, occurred. Furthermore, homeschooling alters eating habits, including snacking between meals and comfort eating (3, 4). All those changes might have affected the physical health of children with diabetes, including possible weight gain and worsening of metabolic control.

Obesity in children and adolescents is a growing health problem. According to Majcher et al., in the group of 656 Polish children with excess body weight, 21.8% had 2^nd^-degree obesity; furthermore, 84% of those patients were already obese at the age of 6 (5). The increasing prevalence of overweight and obesity is also observed in people with type 1 diabetes (T1D), affecting up to 35% of patients, as well as augmenting the risk of chronic complications of diabetes. In T1D, like in the general population, a high supply of simple carbohydrates and fats in the diet along with sedentary lifestyle and physical inactivity are factors contributing to the increase in the BMI (6). Furthermore, both overweight and physical inactivity in patients with T1D influence beta-cell residual function; therefore, it may negatively impact diabetes remission occurrence and duration (7).

In this regard, the aim of the paper was to analyze whether the restrictions introduced due to the COVID-19 pandemic have influenced the metabolic control of children with T1D, as well as the change in the body mass of those patients.

## Patients and methods

In this retrospective study, we evaluated pediatric patients with T1D from at least 12 months before the first visit, treated by one therapeutic team, who had at least two in-person visits in the outpatient clinic in 2020 (before and 6 months after the lockdown announcement in Poland), as well as at least two in-person visits in 2021 (6 and 12 months following the last visit in 2020). The following data were analyzed: the time in range (TIR) (70–180 mg/dl, %), HbA1c, as well as the total daily dose (TDD) of insulin (IU/day and IU/kg of body mass), height (measured to the nearest 0.1 cm), and weight (measured to the nearest 0.01 kg) from the visit before the announcement of the pandemic restrictions (15 March 2020), during the lockdown (second visit after 6 months in 2020), and within the period of loosened restrictions (two visits in 2021 in 6 months interval). The body mass index (BMI, kg/m^2^) together with BMI percentile (BMI%ile) were calculated. BMI%ile was calculated according to WHO BMI growth charts (8). BMI%ile is a measure of the relative BMI, adjusted for a child’s age and sex. If a child has maintained the BMI over time, BMI%ile would not change despite the difference in the absolute value of the BMI, whereas an increase or decrease in BMI%ile indicates that the BMI gain is greater or less in comparison with the reference sample of peers of the same age and sex. Nutrition status terminology was based on Ref (9). Underweight was defined as BMI < 5^th^ %ile, a normal BMI was recognized when the BMI was within 5^th^ to 85^th^ %ile, overweight was diagnosed if BMI ≥ 85^th^ %ile and < 95^th^ %ile, and obesity if BMI ≥ 95^th^ %ile. Newly diagnosed T1D, complete remission of T1D, and other types of diabetes were the exclusion criteria.

Descriptive statistics were calculated for all four time points. The data were presented as the arithmetic mean (x) and standard deviation (SD) for continuous variables and as percentages for categorical variables. As only patients with complete data from all four visits were included in the study, no data imputation was required. The W Shapiro–Wilk test was used to verify the compliance of the distribution of quantitative variables of the analyzed sample with the normal distribution. To determine if there was a change from one measurement to the other, the paired Student’s t test was used if the distribution of the samples did not differ significantly from the normal distribution with statistically equal variance. If those assumptions were not met, the Wilcoxon signed-rank test was used. To detect differences among multiple dependent samples, repeated-measures ANOVA or Friedman tests were used appropriately, followed by *post hoc* analysis with the Tukey test or Dunn test with Bonferroni correction. In order to evaluate the difference between the BMI category of studied children on each visit, the McNemar test for paired proportions was used, as patients served here as their own control. In all statistical tests, a significance level of α = 0.05 was assumed.

The study was approved by the Bioethical Committee of Wrocław Medical University, Wrocław, Poland (No. KB-487/2022).

## Results

A total of 185 patients with T1D were included in the study (96 boys, 51.9%), aged 2–18 years (11.5 ± 3.5), with disease duration from 1 to 15 years (4.4 ± 3.1). Furthermore, 135 of them (72.9%) use continuous subcutaneous insulin infusion (CSII), and 142 (76.8%) use continuous glucose monitoring (CGM) or flash glucose monitoring (FGM) systems. Boys and girls did not differ according to age or duration of the disease (p=0.8025 and p=0.6411, respectively).

**Figure 1:**
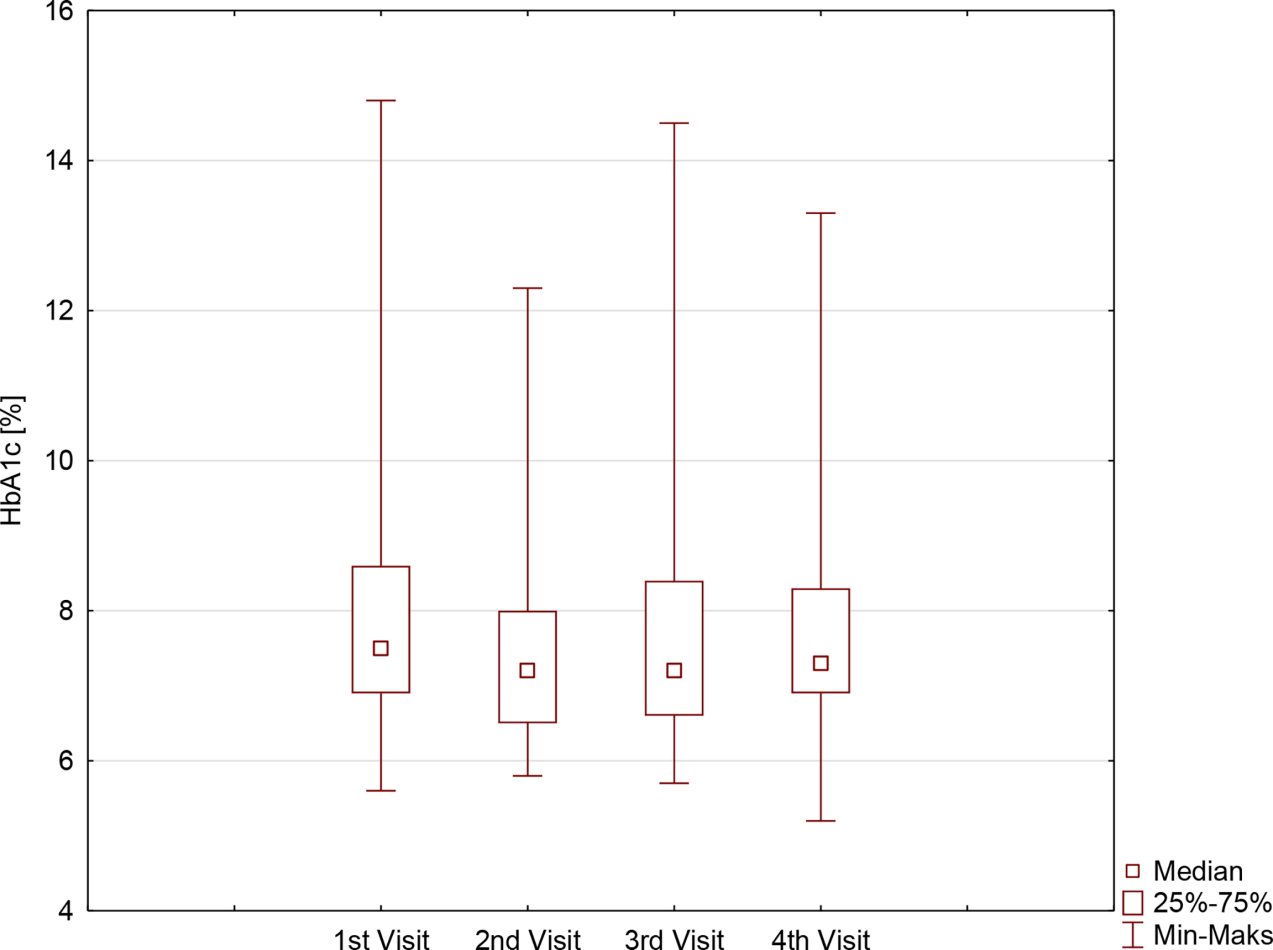
HbA1c [%] in children with type 1 diabetes before and during the SARS-CoV-2 pandemic.

During the first months of the studied period, despite comparable TIR (57.5 ± 21.4% vs. 59.9 ± 20.5%, p=0.3604), a significant improvement in HbA1c was noticed: 7.9 ± 1.6% vs. 7.5 ± 1.4%, (p=0.0336). It was followed by the stabilization of metabolic control, with comparable results regarding both TIR and HbA1c on second, third, and fourth visits: 59.9 ± 20.5% vs. 63.8 ± 17.6% vs. 63.1 ± 16.4%, p=0.4796, and 7.5 ± 1.4% vs. 7.6 ± 1.3% vs. 7.7 ± 1.3%, p=0.6065, respectively. Those observations were comparable for both sexes, although the HbA1c concentration on the last visit in girls was significantly lower compared with the HbA1c concentration in boys (**Table 1**, **Figure 1**). Furthermore, the TIR on the second and third visits was significantly lower in girls than in boys.

**Table 1:** Metabolic control in children with type 1 diabetes before and during SARS-CoV-2 pandemic according to sex.

		Total	Boys	Girls	*P* value
Visit 1	HbA1c [%]	7.9 ± 1.6	7.8 ± 1.8	8.0 ± 1.4	0.7458
	TIR [%]	57.5 ± 21.4	61.4 ± 21.3	51.4 ± 20.0	0.1007
Visit 2	HbA1c [%]	7.5 ± 1.4	8.0 ± 1.9	7.6 ± 1.4	0.1070
	TIR [%]	59.9 ± 20.5	60.5 ± 20.4	52.8 ± 21.2	0.0127
Visit 3	HbA1c [%]	7.6 ± 1.3	7.8 ± 1.7	7.4 ± 1.0	0.0550
	TIR [%]	63.8 ± 17.6	68.5 ± 16.7	58.4 ± 17.6	0.0001
Visit 4	HbA1c [%]	7.7 ± 1.3	7.9 ± 1.4	7.5 ± 1.0	0.0276
	TIR [%]	63.1 ± 16.4	63.9 ± 16.2	62.1 ± 17.0	0.4618
*P* value *(ANOVA)*	HbA1c	0.0678	0.7654	0.2979	
	TIR	0.8363	0.3324	0.0952	

TIR, time in range, 70–180 mg/dl.

During the whole study period, the TDD increased significantly (p=0.0003) from 37.3 ± 18.9 units/day on the first visit to 41.7 ± 21.1 units/day on the second visit (p=0.0353) up to 46.8 ± 22.7 units/day on the fourth visit (p=0.0258); however, insulin demand related to body mass remained constant (0.8 ± 0.2 units/kg on the first visit vs. 0.8 ± 0.3 units/kg on the last visit, p=0.4857). Likewise, the proportion of basal insulin in the TDD was comparable throughout the observation (39.7 ± 11.3% on the first visit vs. 39.3 ± 13.6% on the last visit, p=0.7428). Those findings were similar for both sexes (p>0.05).

**Figure 2:**
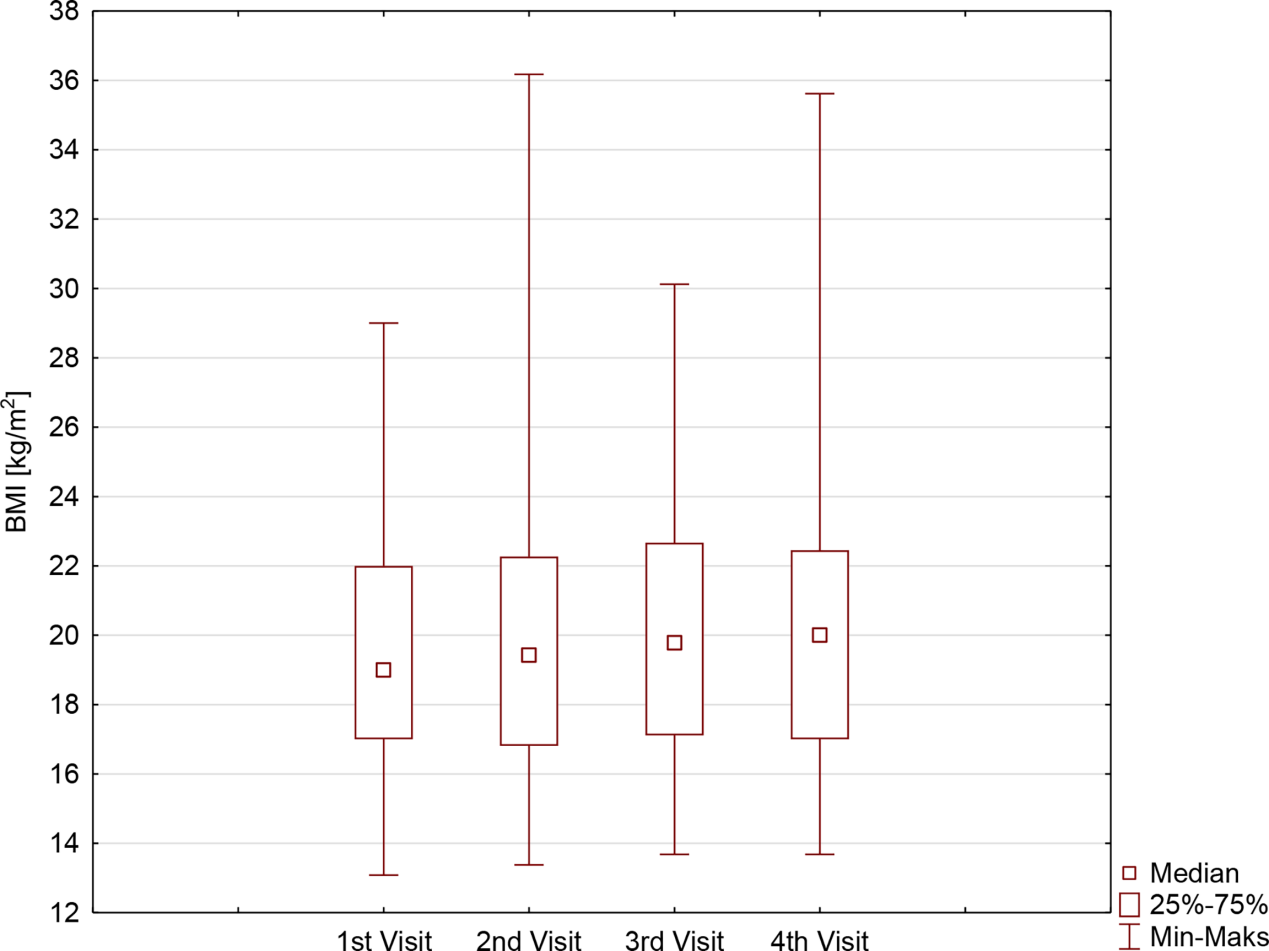
The body mass index (BMI) [kg/m^2^] in patients with type 1 diabetes before and during SARS-CoV-2 pandemic.

The BMI, as well as BMI%ile, of the examined patients increased significantly during the study period (p=0.0027 and p=0.0001, respectively), especially in the first months of restrictive lockdown (from 19.1 ± 3.7 kg/m^2^ before lockdown vs. 20.1 ± 3.6 kg/m^2^ on the second visit, p<0.0001, and from 58.9 ± 26.2 before lockdown vs. 64.6 ± 26.0 on the second visit, p<0.0001), stabilizing when the pandemic limitations were loosened (20.4 ± 3.9 kg/m^2^ on the third visit vs. 20.9 ± 4.1 kg/m^2^ on the last visit, p=0.2302, and 60.7 ± 26.6 kg/m^2^ on the third visit vs. 58.1 ± 28.4 kg/m^2^ on the last visit p=0.4098). It is noteworthy that a significant increase in BMI was observed in girls but not in boys, with a clear difference on the last visit. BMI%ile, however, changed during the study period regardless of sex (**Table 2**, **Figure 2**), overall indicating significant weight gain in girls and reduction of body mass in boys.

**Table 2:** Body mass index in type 1 diabetic children before and during SARS-CoV-2 pandemic according to sex.

		Total	Boys	Girls	*P* value
Visit 1	BMI [kg/m^2^]	19.1 ± 3.7	18.9 ± 3.4	19.3 ± 4.0	0.6043
	BMI%ile	58.9 ± 26.2	64.8 ± 23.6	52.5 ± 27.7	0.0417
Visit 2	BMI [kg/m^2^]	20.1 ± 3.6	18.8 ± 2.7	19.4 ± 3.9	0.2225
	BMI%ile	64.6 ± 26.0	64.3 ± 26.1	64.9 ± 26.3	0.9295
Visit 3	BMI [kg/m^2^]	20.4 ± 3.9	20.1 ± 4.2	20.8 ± 3.4	0.2165
	BMI%ile	60.7 ± 26.6	60.2 ± 27.1	61.3 ± 26.4	0.8532
Visit 4	BMI [kg/m^2^]	20.9 ± 4.1	20.3 ± 4.5	21.6 ± 3.5	0.0304
	BMI%ile	58.1 ± 28.4	57.8 ± 31.1	58.5 ± 25.5	0.8858
*P* value *(ANOVA)*	BMI [kg/m^2^]	p=0.0027	0.0956	0.0220	
	BMI%ile	0.0001	0.0009	0.0019	

BMI, body mass index; BMI%ile, body mass index percentile.

**Figure 3:**
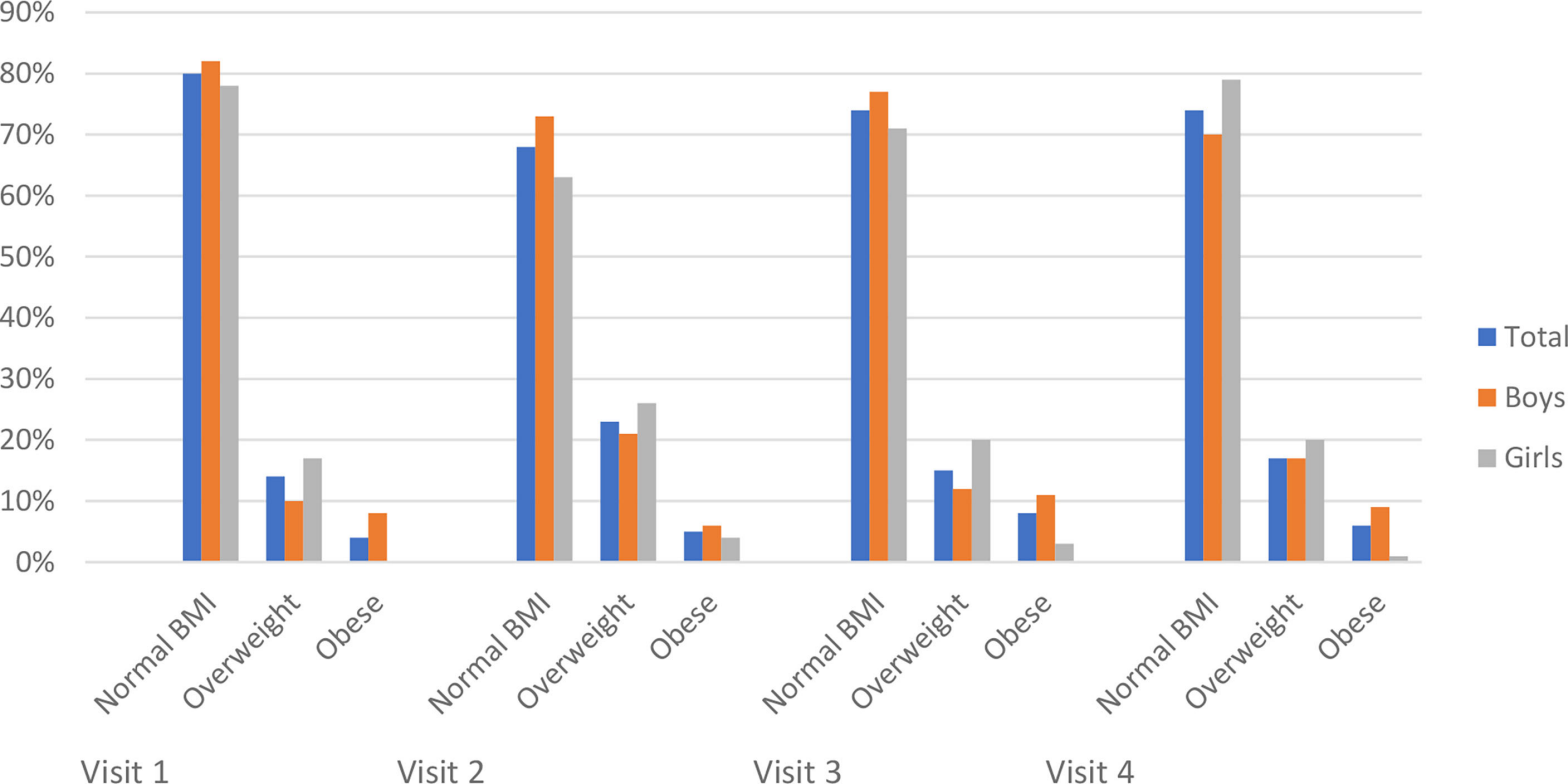
The prevalence of overweight and obesity in children with type 1 diabetes before and during SARS-CoV-2 pandemic.

The prevalence of overweight and obesity in the studied population is presented in **Table 3** and **Figure 3**. The overall proportion of different categories of BMI was stable (p>0.05) throughout the study period; however, there was a significant change in the percentage of patients with overweight and obesity, with an increase during the lockdown period from 18% on visit 1 to 28% on visit 2 (p<0.0001), and the reduction to 23% on visit 3 (p<0.0001), stabilizing afterward to 23% on visits 3 and 4 (p>0.05). Finally, the percentage of patients with excess body mass increased during the observation by 5.4% (**Table 4**).

**Table 3:** The prevalence of underweight, overweight, and obesity in children with type 1 diabetes before and during SARS-CoV-2 pandemic.

	BMI criterion*	Total	Boys	Girls	*P* value
Visit 1	Underweight	3%	0%	6%	0.1280
	Normal BMI	80%	82%	78%	0.6690
	Overweight	14%	10%	17%	0.3803
	Obese	4%	8%	0%	0.0852
Visit 2	Underweight	3%	0%	7%	0.1257
	Normal BMI	68%	73%	63%	0.4107
	Overweight	23%	21%	26%	0.6510
	Obese	5%	6%	4%	0.7283
Visit 3	Underweight	3%	0%	6%	0.1446
	Normal BMI	74%	77%	71%	0.5812
	Overweight	15%	12%	20%	0.3770
	Obese	8%	11%	3%	0.2143
Visit 4	Underweight	2%	4%	0%	0.0992
	Normal BMI	74%	70%	79%	0.2238
	Overweight	17%	17%	20%	0.6475
	Obese	6%	9%	1%	0.0332
*P* value		0.9905	0.7687	0.8425	

* Underweight (BMI < 5^th^ %ile); normal BMI (BMI 5^th^ - 85^th^ %ile); overweight (BMI ≥ 85^th^ %ile – 95^th^ %ile); obese (BMI ≥ 95^th^ %ile) (9). BMI, body mass index.

**Table 4:** The difference between paired proportions of patients with overweight and obesity on each visit.

Visit interval	Difference	95% Confidence Interval	*P* value (McNemar test)
Visit 1–Visit 2	10.3%	5.9% to 14.6%	<0.0001
Visit 2–Visit 3	-4.6%	-7.6% to -1.7%	<0.0001
Visit 3–Visit 4	0%	0%	
Visit 1–Visit 4	5.4%	2.1% to 8.7%	<0.0001

Lastly, we evaluated whether overweight and obesity at the beginning of the observation influenced the change in body mass during the study period. The difference between the BMI on the first and last visits was significantly (p=0.0078) greater in patients who had excess body mass before the pandemic (-1.8 ± 4.8 kg/m^2^) than in children with normal body weight (0.3 ± 2.2 kg/m^2^). On the other hand, those patients reduced their BMI%ile by 0.1 ± 7.0 (p=0.0046), whereas children without excess body mass gained weight and their BMI%ile increased by 1.9 ± 8.7 (p=0.0002). The difference was not significant between those groups (p=0.2554).

## Discussion

Rundle et al. hypothesized that restrictive mitigation measures due to the COVID-19 pandemic will exacerbate the risk factors of weight gain in children mainly by increasing food insecurity along with higher consumption of ultraprocessed and calorie-dense food. Consequently, children will experience higher calorie diets. Furthermore, social distancing and “stay at home” recommendations reduce opportunities for physical activities and enhance sedentary behaviors and screen time (10). Observations done during the early months of the COVID-19 pandemic by Pietrobelli et al. support Rundle’s hypothesis. In this small study on lifestyle changes during lockdown in 41 obese children, the authors found an increased number in meals eaten per day followed by an increased intake in potato chips, sugary drinks, and red meat. Sleep time and screen time increased significantly, whereas sports time decreased significantly. Although post-confinement measurements of body mass were not performed in this study due to pandemic limitations, it may be assumed that the above-mentioned negative lifestyle changes will result in an excess weight gain during the lockdown (4).

Obviously, a negative association of mitigation measures related to the pandemic with reduced activity level, increased sedentary behavior, and weight gain is observed not only in the general population but also in people with diabetes. In a cohort of children with T1D, we have observed a significant increase in BMI, as well as BMI%ile, during the study period. Weight gain was especially distinct in the first months of restrictive lockdown; nevertheless, when pandemic mitigations were loosened, both the BMI and BMI%ile stabilized. Moreover, we have found that the prevalence of overweight and obesity increased during the study period by 5.4%; however, the greatest augmentation, reaching 10.3%, happened during lockdown. Our results are in line with Weaver et al.’s observation that COVID-19 led to an accelerated increase in the BMI z-score gain. This interesting investigation was designed as an interrupted time-series study with anthropometric data collected in August/September from 2017 to 2020, followed by an estimation of mixed-effects linear regression of the yearly BMI z-score change before and during the COVID-19 pandemic (i.e., 2017–2019 and 2019–2020, respectively). It was found that the non-pandemic yearly BMI z-score change was stable, reaching +0.03 every year (95% CI=-0.10, 0.15; difference 0.0, 95% CI=-0.09, 0.08), whereas during the pandemic year, it was +0.34 (95% CI=0.21, 0.47), with an acceleration in the BMI z-score change of +0.31 (95% CI=1.19, 0.44). The increase in the BMI z-score during the COVID-19 pandemic corresponded with an increased risk of excess adiposity in those children (odds ratio 1.80, 95% CI=1.40, 2.33) (11). Similarly, Jarnig et al.’s study showed that in a cohort of 764 Austrian children, the implementation of strict pandemic limitations was associated with an increase in the BMI z-score by 0.16 ± 1.10 during 1 year of observation. In this study, the percentage of children with excess adiposity increased by 3.8%, from 20.3% to 24.1% (12).

One explanation of the observed weight gain, as mentioned by Pietrobelli et al. and Rundle et al., is the unhealthy change in eating behavior, including the increased consumption of ultraprocessed, calorie-dense comfort foods (4, 10). As the TDD increased during the observation, we may suspect in the studied cohort of an increased caloric intake during the pandemic; however, regrettably, it was not evaluated in a direct manner. Still, downloads of personal insulin pump data revealed a higher number of meal boluses during lockdown (data not shown). Moreover, in some children, an increased basal rate during the day might have covered snacking. Increased calorie intake is apparently not the whole story. The other part is the decrease in physical activity, as school closures and social distancing orders issued across the world reduced the opportunities to exercise (4, 10). Restrictions imposed in many countries, including Poland, resulted in limited occasions to be physically active. The negative association of pandemic mitigation measures with the level of physical activity and sedentary behavior, and subsequent decreased physical fitness, was described in numerous studies and is considered to be the indirect consequence of the COVID-19 pandemic (4, 12–16). Unfortunately, in our study, we did not measure the level of physical activity, which is undoubtedly a limitation of the study. It has to be underlined, however, that the initial COVID-19 mitigation measures implemented in Poland were rigorous and included the closure of not only gyms and swimming pools, but also playgrounds and parks, as well as prohibiting children under the age of 16 to stay outside their home without adult supervision. Together with physical education classes, all other organized sport activities were suspended. Therefore, we may assume that weight gain found in our study, at least in part, was the result of reduced activity. Pandemic mitigation measures implemented in Poland were comparable to those imposed in Austria. Hence, Jarnig et al.’s study, which examined the association of COVID-19 restrictions with changes in cardiorespiratory fitness (CRF) in primary schoolchildren, could be referred to the situation in our country and in our cohort (12). They found that, along with weight gain, the CRF SD scores decreased (-1.06, 95% CI=-1.13, -1.0) apparently due to lower physical activity, especially those of higher intensity (12, 17, 18). The increased TDD of insulin, noticed in our study during the lockdown period, may also be the indirect evidence of reduced physical activity, since in children who exercise, a significant reduction in insulin dose is observed and recommended (19–21).

It was assumed that overweight and obese patients tend to gain more weight than subjects with normal body mass. The explanation for this observation seems to be simple: people with excessive adiposity more often present unhealthy dietary habits, including increased consumption of sweets, meat, and fried food, while their physical activity is lower (3, 22). Indeed, the increased quantity and decreased quality of meals were confirmed in multicenter studies to be associated with a higher BMI (23). The survey from Poland revealed that obese subjects had the lowest frequency of vegetables, fruits, and legumes consumption on a daily basis (58.5% and 13.8%, respectively), whereas everyday consumption of fast foods and meat was the highest (3.2% and 40.4%, respectively). The weight gain in overweight and obese people was the greatest. The authors conclude that mitigation measures may amplify preexisting excess adiposity and, therefore, magnify health issues related to body mass (24). The meta-analysis by Bakaloudi et al. confirmed that there was a tendency toward an increase in body weight during lockdown in the majority of studies and populations (25).

The meta-analysis by Tu-Hsuan Chang et al. showed that there was an increase in the BMI of children in the general population (MD 0.77, 95% CI 0.33–1.20;p= 0.0006), associated with the period with strict restrictions, followed by the increased rates of obesity (OR 1.23, 95% CI 1.10-1.37; p = 0.0002) and overweight (OR 1.17, 95% CI 1.06-1.29; p = 0.001). The authors performed the subgroup analysis according to the study population, which showed that the BMI did not change significantly during the lockdown both in children with T1D mellitus and obesity. It has to be underlined though that the substantial heterogeneity among studies, also in the subgroup analysis, was reported (26). On the contrary, in our study, the BMI as well as BMI%ile of pediatric patients with T1D increased significantly during the study period, especially in the period of restrictive lockdown, stabilizing subsequently with the withdrawal of the pandemic limitations. On the other hand, in overweight and obese patients with T1D, in absolute values, the change of the BMI was significantly higher in comparison with normal-weight children; however, overweight and obese subjects reduced their BMI%ile during lockdown. Thus, it can be concluded that children who had excess adiposity before the COVID-19 pandemic did not experience accelerated weight gain during the pandemic. Comparable results were reported by Weaver et al., who revealed that the BMI z-score gain increase was enhanced during the pandemic lockdown, but this observation did not apply to pre-pandemic overweight and obese children (11). We may only speculate that improved parental control while being together during the lockdown, and less possibility to buy unhealthy snacks, resulted in less weight gain.

The question is whether the observed changes in body weight could be transitory once the imposed restrictions are withdrawn. Matsumoto et al. found that in obese patients, a decrease in body fat was significantly higher during lockdown in comparison with the group unaffected by mobility restrictions. On the other hand, their exercise tolerance (VO2 at anaerobic threshold and peak VO2) was significantly lower (27). This corresponds with Jarnig et al.’s study in children, who observed reduced CRF due to reduced physical activity during lockdown (12). The time after COVID-19 mitigations were withdrawn worldwide is too short to be sure whether children will recover from the consequences of the previous restrictions. Our study brings some data on this. As in many other studies, we have observed an increase in the BMI and BMI%ile during the period of restrictive lockdown. It resulted in an increase in the prevalence of overweight and obesity in the studied cohort by approximately 10%, from the initial 18% on the pre-pandemic visit to 28% on the second visit (the lockdown visit). Fortunately, a further increase in both the BMI and BMI%ile was not observed, indicating the stabilization of patients’ body mass when pandemic mitigations were loosened and the intensification of physical activity was possible. Furthermore, the prevalence of excess adiposity was reduced by 4.6% between the second and third visits, giving the overall increase in overweight and obesity rate by 5.4% throughout the study period. It clearly indicates the importance of physical activity in maintaining body mass (17, 18).

The deterioration of metabolic control may be expected in patients with T1D as a result of lower intensity of physical activity, negative change in nutritional habits, and an increased level of stress accompanying the sudden change of lifestyle due to lockdown and separation anxiety. Indeed, people with diabetes who reported deterioration in metabolic control experienced higher stress, followed by increased insulin demand during lockdown, as reported by Ruissen et al., but, surprisingly, an elevated level of anxiety was not associated with HbA1c itself. Furthermore, in this study, 40.9% of patients reported weight gain and 45.7% decreased physical activity without any difference between people with type 1 and type 2 diabetes, and, again, without any influence on HbA1c. Ruissen et al. showed in people with T1D that HbA1c was lower during lockdown than before this period (7.52 ± 1.1% *vs*. 7.68 ± 1.2%, p<0.0001), even though the mean difference is not clinically relevant. Additionally, glucose monitoring data showed higher time in range (63.4% *vs*. 60.5%, p=0.0009) together with lower time above range (32.1% *vs*. 34.6%, p<0.003), but glucose variability did not change. The authors concluded that the improvement in metabolic control was a consequence of more focus on diabetes self-management, indicated by more frequent glucose monitoring, as the number of FGM scans per day increased from 9.6 ± 6.5 before lockdown to 11.8 *vs*. 8.1 during lockdown (p<0.01). Interestingly, the biggest improvement in HbA1c was reported in patients in the highest pre-lockdown tertile of HbA1c (28). Continuous glucose monitoring systems significantly improved the metabolic control of diabetes (29). Similar results were observed in our cohort, where HbA1c lowered by 0.4% during the lockdown, despite comparable time in range. The following visits showed the stabilization of improvement in the metabolic control achieved during the first months of the studied period. Our results are in agreement with an Italian report on 62 patients, showing the time in range increased by 3% (p=0.008), resulting in the improvement from 7.4% to 7.25% of the glucose management indicator during the first 3 months of lockdown. Furthermore, glucose variability lowered as glucose standard deviation and coefficient of variation improved across the study (p<0.0001 and p=0.001, respectively) (30). Another Italian study revealed the dependence of metabolic results on the patient’s age because CGM metrics improved during the lockdown in children (glucose standard deviation, p=0.029 and time below range, p=0.029) and adults (time in range, p<0.001), whereas, in teenagers, CGM metrics remained unchanged during lockdown. Furthermore, it was reported that adult patients who improved metabolic control were more physically active and younger, whereas those who worsened glucose control showed higher perceived stress compared with others (31). Eberle and Stichling in their systematic review, which included 33 studies with 2,881 T1D participants and 1,823 type 2 diabetes participants, concluded that the glycemic control of patients with T1D improved significantly during lockdown (32). The authors of the above studies suggest that continuous parental management associated with positive changes in self-management of the disease could have beneficial effects on metabolic control in T1D. The role of telemedicine was also underlined (30–32). The results from the Diabetes Prospective follow-up registry regarding metabolic control during the COVID-19 pandemic lockdown in a large cohort of German children with T1D showed comparable metabolic control before, during, and after lockdown in the spring months of 2020. The authors aggregated HbA1c values from laboratory measurements and, from continuous glucose monitoring, derived estimates into a combined glucose indicator (CGI), which is analogous to HbA1c itself. The CGI values of 19,729 patients for 2020 were insignificantly higher compared with those for 2019, whereas the time in range increased and the mean sensor glucose decreased in 2020. It is noteworthy that there were fewer hospitalizations in 2020 in comparison with 2019 (33). This is in line with the observation of our center (data not shown). Stable metabolic control during the period of COVID-19 related mitigation measures was also found in pediatric patients with T1D from Greece and Israel (34, 35). To our knowledge, no study has indicated a deterioration of metabolic control in patients with T1D. Again, the “stay at home” rule may have had a positive effect on metabolic control in diabetic patients by increasing the time spent under parental supervision and parental diabetes management. Furthermore, slowdown in daily activities might have resulted in focusing on one’s diabetes management, whereas spending more time in a private environment reduced the embarrassment in performing diabetes care with others present and the diabetes-related stigma, especially in adolescents (36, 37).

Undoubtedly, the COVID-19 pandemic and necessary restrictions resulting from it have worsened the burden of childhood obesity. Furthermore, the reduction of physical activity along with the increase in sedentary behavior not only negatively influenced changes in the BMI but also impaired relevant health-related parameters including cardiorespiratory fitness (CRF). The CRF level is negatively correlated with markers of adiposity: BMI, waist circumference, and quantity of body fat. The higher the level of CRF during childhood, the lower the risk factors of metabolic syndrome and cardiovascular disease (CVD) later in life (38). The role of diabetes as a cardiovascular risk factor is well known. Fortunately, restrictions related to COVID-19 generally did not impair metabolic control in people with T1D (39–41). Treatment of CVD risk factors, including excess adiposity, as well as regular physical activity plays an important role in the primary and secondary prevention of CVD (42). This observation is true not only in the general population but also in people with diabetes (43). Therefore, it could be assumed that increasing obesity and worsening of CRF due to prolonged periods of forced inactivity will likely have additional implications for the health of children and adolescents with diabetes. Hence, interventions to ensure the recovery to an age-adequate BMI and CRF level are needed and should be undertaken (44).

## Conclusions

The parameters of metabolic control in pediatric patients with T1D during the pandemic period remained stable; however, weight gain and an increase in daily insulin dose have been observed, possibly due to reduced physical activity.

## Data availability statement

The raw data supporting the conclusions of this article will be made available by the authors, without undue reservation.

## Ethics statement

This study was reviewed and approved by The study was approved by the Bioethical Committee of Wrocław Medical University, Wrocław, Poland (No. KB-487/2022). Written informed consent from the participants’ legal guardian/next of kin was not required to participate in this study in accordance with the national legislation and institutional requirements.

## Author contributions

AZ-K: Conceptualization; Formal analysis; Investigation; Methodology; Project administration; Validation; Writing - original draft, review & editing. BW: Investigation; Methodology; Validation; Writing - review & editing. AN: Conceptualization; Methodology; Supervision; Writing - review & editing. All authors contributed to the article and approved the submitted version.

## Funding

The publication fee for this article was paid by the Medical University of Wroclaw.

## Conflict of interest

The authors declare that the research was conducted in the absence of any commercial or financial relationships that could be construed as a potential conflict of interest.

## Publisher’s note

All claims expressed in this article are solely those of the authors and do not necessarily represent those of their affiliated organizations, or those of the publisher, the editors and the reviewers. Any product that may be evaluated in this article, or claim that may be made by its manufacturer, is not guaranteed or endorsed by the publisher.
